# Assessing efficacy of ADCY10 inhibitors in cervical mucus

**DOI:** 10.1093/molehr/gaag035

**Published:** 2026-06-04

**Authors:** Justine Fischoeder, Jai G Marathe, Deborah J Anderson, Lonny R Levin, Jochen Buck, Carla Ritagliati

**Affiliations:** Department of Pharmacology, Weill Cornell Medicine, New York, NY, USA; Department of Medicine, Boston University Chobanian & Avedisian School of Medicine, Boston, MA, USA; Department of Medicine, Boston University Chobanian & Avedisian School of Medicine, Boston, MA, USA; Department of Pharmacology, Weill Cornell Medicine, New York, NY, USA; Department of Pharmacology, Weill Cornell Medicine, New York, NY, USA; Department of Pharmacology, Weill Cornell Medicine, New York, NY, USA

**Keywords:** human sperm, sAC, contraceptive, cervical mucus, viscoelasticity, female reproductive tract, capillary assay

## Abstract

Male fertility depends on sperm motility, which enables sperm to traverse the female reproductive tract to the site of fertilization. Soluble adenylyl cyclase (sAC) is a key regulator of sperm motility, defining it as a promising non-hormonal contraceptive target for women and men. To evaluate the efficacy of sAC inhibitors under conditions mimicking the female reproductive tract, we employed a capillary assay using cervical mucus isolated from humans or cows as well as methylcellulose, a viscoelastic liquid that simulates cervical mucus. Addition of a sAC inhibitor, either directly to semen, modeling an on-demand male contraceptive, or to the cervical mucus, approximating a female contraceptive, effectively blocked sperm migration. These studies provide proof-of-concept for the efficacy of sAC inhibitors under physiologically relevant conditions.

## Introduction

Motility of ejaculated spermatozoa is considered the most predictive parameter of their functional capacity and male fertility (Mortimer *et al.*, 1984). Sperm are produced in the testis and stored in a dormant state awaiting ejaculation in the cauda epididymides, an environment characterized by low pH and reduced bicarbonate levels. At ejaculation, sperm are mixed with seminal fluid and exposed to the mucus-lined female reproductive tract, which increases intracellular bicarbonate concentrations, stimulating the bicarbonate-regulated soluble adenylyl cyclase (ADCY10; sAC) to produce the second messenger cAMP and initiate sperm motility and capacitation (Chen *et al.*, 2000; Litvin *et al.*, 2003; Esposito *et al.*, 2004; Hess *et al.*, 2005; Xie *et al.*, 2006; Buffone *et al.*, 2014; Balbach *et al.*, 2021). Sperm motility is absolutely dependent upon active sAC because in both humans (Akbari *et al.*, 2019; Zeb *et al.*, 2024) and mice (Esposito *et al.*, 2004; Hess *et al.*, 2005; Ayoub *et al.*, 2025), mutations that disrupt sAC activity result in immotile sperm and male-specific sterility. Because men devoid of sAC exhibit no other acute phenotypes, we posited that a fast-acting sAC-specific inhibitor could provide on-demand male contraception, where a man taking a sAC inhibitor shortly before intercourse would be temporarily infertile. We previously leveraged a sAC-specific inhibitor with long-residence time on sAC protein to prove this principle in mice. Male mice treated with the sAC inhibitor TDI-11861 were infertile for 2.5 h following treatment (Balbach *et al.*, 2023). By the following day, treated mice were once again fully fertile.

Ejaculated semen transiently neutralizes the normally acidic vagina, making it temporarily hospitable to sperm. Because the vagina reacidifies following intercourse, there is a brief window for motile sperm to traverse the mucus-filled cervix to escape into the supportive environment of the uterus. Both sperm motility and cervical mucus penetration are key determinants of fertilizing potential, with poor penetration reflecting reduced sperm function and lower pregnancy rates (Eggert-Kruse *et al.*, 1989). To navigate through the cervix, uterus, and fallopian tubes to reach the oocyte, sperm acquire an asymmetric, high-amplitude flagellar movement termed hyperactivated motility. In addition to being required for basal motility (Esposito *et al.*, 2004; Hess *et al.*, 2005; Akbari *et al.*, 2019; Ayoub *et al.*, 2025; Zeb *et al.*, 2024), sAC is necessary for the transition to hyperactivated motility, which has been proposed to be critical for sperm to escape the vagina, detach from the oviductal epithelium, penetrate the cumulus and zona pellucida, and sustain motility through the viscoelastic fluids that line the female reproductive tract (Mortimer *et al.*, 1986; Suarez *et al.*, 1991; Pérez-Cerezales *et al.*, 2016; Ritagliati *et al.*, 2025).

Male fertility is often studied *in vitro* by examining semen parameters such as sperm concentration, morphology, and motility patterns measured via computer-assisted sperm analysis (CASA). The conditions used for studying sperm motility via CASA are optimized for supporting IVF. To support our efforts to develop sAC inhibitors into contraceptives, we sought a physiologically relevant method for measuring the motility of unprocessed ejaculated sperm through viscoelastic solutions. Capillary penetration methods were created to study how sperm behave in environments that mimic the female reproductive tract (Katz *et al.*, 1980; Pandya *et al.*, 1986; Ivic *et al.*, 2002). These employ cervical mucus or viscoelastic agents such as hyaluronic acid, methylcellulose (MC), or polyvinylpyrrolidone that mimic the viscoelasticity of cervical mucus (Ivic *et al.*, 2002). The most relevant viscoelastic medium for our efforts to develop a male or female contraceptive is human cervical mucus (HCM). However, because HCM is a limited resource, we also tested migration in bovine cervical mucus (BCM), which is commercially available but could be subject to variabilities inherent in fluids derived from animals, and 1% MC, which is the least physiologically relevant but is well defined, highly reproducible, and validated as an alternative (Ivic *et al.*, 2002). Here, we asked whether sAC inhibitors are effective at blocking sperm migration through viscoelastic fluids in capillary penetration assays, and we tested whether these assays can be employed following minimal manipulation of undiluted semen. A goal of these studies is to determine whether capillary migration can be leveraged as an additional biomarker reflecting drug biodistribution into sperm and/or semen during clinical trials of sAC inhibitor on-demand male contraceptives.

## Materials and methods

### Human participants

This study conformed with the Declaration of Helsinki and was approved by Weill Cornell Medicine’s Institutional Review Board (IRB 21-03023495) & Boston University’s Medical Campus’ IRB (IRB H-41454). All human participants signed written informed IRB-approved consent forms.

### Human sample preparation

Human semen samples were collected from healthy donors. Only ejaculates that satisfied the WHO 2010 standards for normal semen quality (volume ≥1.5 ml, sperm concentration ≥15 million/ml, total motility ≥40%, progressive motility ≥32%, and normal morphology ≥4%) were included. After collection, samples were allowed to liquefy for 30–60 min at 37°C. The semen was then either used directly for experiments or processed through density-gradient centrifugation with Isolate medium (Irvine Scientific, Santa Ana, CA, USA) according to the manufacturer’s guidelines. Purified sperm were washed and resuspended in modified human tubal fluid (HTF), composed of 101.5 mM NaCl, 4.8 mM KCl, 0.2 mM MgSO_4_, 0.37 mM KH_2_PO_4_, 2.04 mM CaCl_2_, 2.78 mM glucose, 0.33 mM sodium pyruvate, 21.4 mM sodium lactate, and 20 mM HEPES, with the pH adjusted to 7.3–7.4 at 37°C using NaOH. All reagents were purchased from Sigma-Aldrich (St. Louis, MO, USA). Sample purity and sperm viability were evaluated by light microscopy, and cell counts were obtained using a hemocytometer. For capacitation, semen or isolated sperm were incubated in HTF supplemented with 25 mM NaHCO_3_ and 3 mg/ml human serum albumin (HSA) (Irvine Scientific).

Midcycle ovulatory cervical mucus was collected from reproductive-aged women (18–45 years) with regular menstrual cycles using an endocervical pipelle (Aspirette Endocervical Pipelle, Cooper Surgical, Trumbull, CT, USA). Participants were not using any hormonal birth control, refrained from intercourse for at least 48 h prior to collection, and had no history of recent pregnancy (within 3 months), douching within 48 h, chronic antibiotic use, or history of genitourinary malignancy. Ovulation was estimated using an online calculator and confirmed with a digital ovulation test (Clearblue Digital Ovulation Predictor Kit, Clearblue, Nashville, TN, USA). The cervical mucus samples were obtained under direct visualization using an unlubricated vaginal speculum and samples within 48 h of a positive ovulation test, stored at 4°C, and used within 3 days. Cervical mucus was diluted at a 1:3 ratio of mucus:phosphate-buffered saline and shaken at 37°C for an hour before use (see Dohadwala *et al.*, 2025). Dilution was necessary to allow the mucus to be aspirated into the needle.

### Computer-assisted sperm analysis

**Figure 1.**
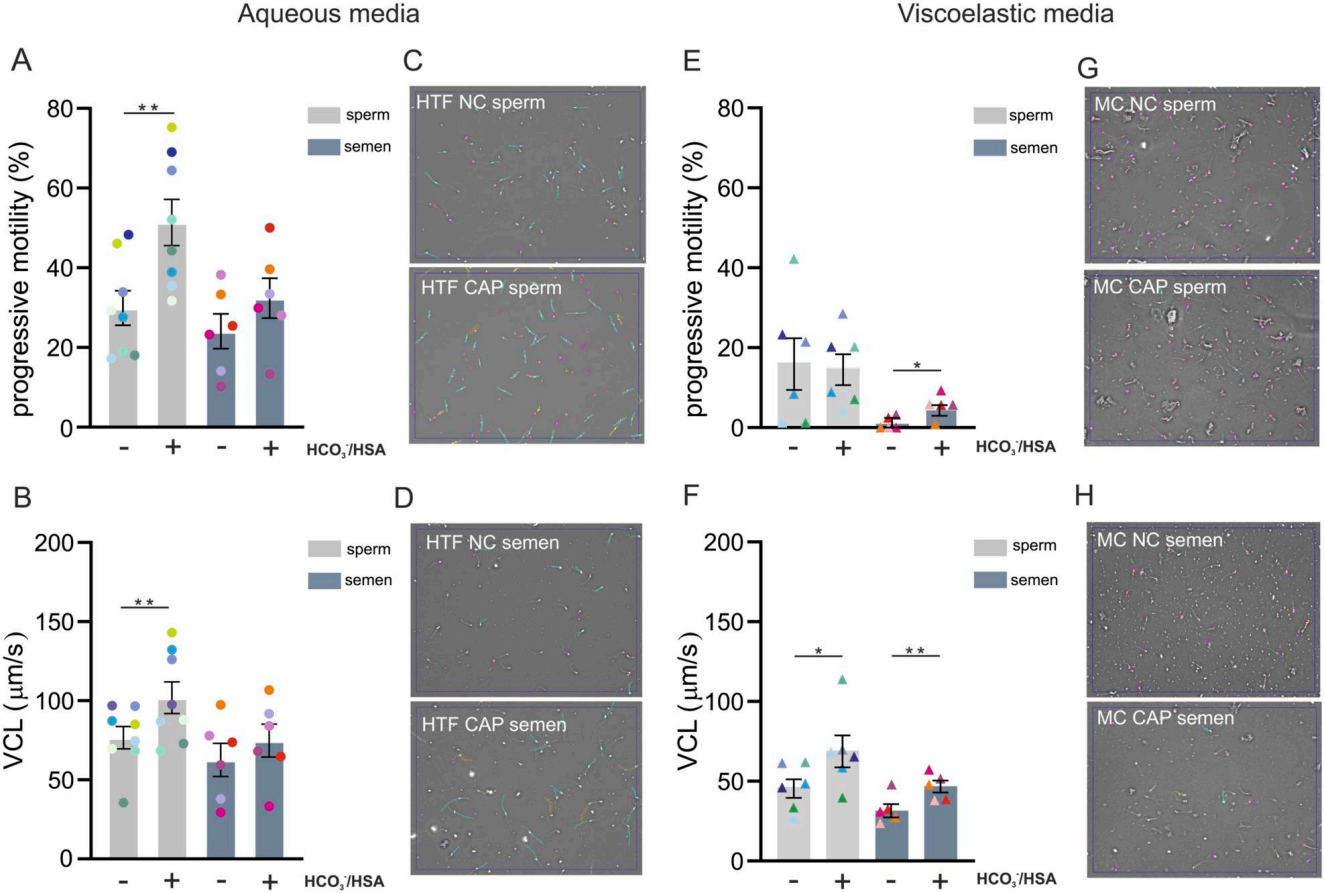
**Sperm motility in computer-assisted sperm analysis (CASA).** Sperm/semen from human donors were diluted in human tubal fluid (HTF) (±methylcellulose (MC)) under non-capacitating (NC) (−bicarbonate, human serum albumin (HSA)) or capacitating (CAP) (+bicarbonate, HSA) conditions. (**A**) The percentage of progressive sperm in sperm/semen; (**B**) the curvilinear velocity (VCL) in sperm/semen. (**E**) The percentage of progressive sperm in sperm/semen in 1% MC. (**F**) The velocity (VCL) in sperm/semen in 1% MC. (**C**), (**D**), (**G**), and (**H**) Exemplary screenshots of CASA recordings for all measured conditions. Track’s color code: motile (green), progressive (turquoise), hyperactivated (orange), slow (pink), static (red). Data in the bar graphs are shown as mean ± SEM, representative of at least five independent experiments (different ejaculate samples), with the individual values for each biological replicate (sperm/semen sample) indicated in different colors. *P* value: *P < 0.05, **P < 0.01 (paired *t*-test/Wilcoxon-rank-test).

Purified human sperm or semen were used at a final concentration of 5–10 × 10^6^ sperm/ml in HTF with or without NaHCO_3_ and HSA (Irvine Scientific). To mimic cervical mucus, 1% MC (4000 cp, Sigma-Aldrich) was added. Sperm/semen samples (6 μl) from each condition were placed into 20-micron Leja standard count two-chamber slides, and a minimum of 200 cells across five fields were counted. CASA was performed with a Hamilton–Thorne digital image analyzer (HTR-IVOS II, Version 1.16.2 Hamilton Thorne Research, Beverly, MA, USA). Progressive motility was defined as average path velocity >25 µm/s and straightness (STR) >80%. Hyperactivated motility was defined as curvilinear velocity (VCL) ≥ 150 μm/s, amplitude of lateral head displacement (ALH) ≥5 μm, and linearity (LIN) ≤50% at 60 Hz. Both progressive and hyperactivated motility values were obtained as percentages of the total sperm from the CASA software. Representative screenshots (Fig. 1) and associated videos (Supplementary Videos S1, S2, S3, S4, S5, S6, S7, and S8) from CASA are shown.

### Capillary assay

Flat capillaries (VitroCom, Mountain Lakes, NJ, USA) were prepared by labeling every 0.5 cm. Capillaries were loaded using 1 ml syringes and 26-gauge needles with either undiluted bovine (Innovative Research, Novi, MI, USA) or diluted HCM or 1% MC (4000 cp) (10 mg/ml) mixed with HTF media (with and without bicarbonate and HSA) (Ivic *et al.*, 2002). For the migration assay, semen samples were allowed to liquefy for 30–60 min after collection, and 200 µl were aliquoted into PCR tubes. Media-filled capillaries were inserted (vertically) into the PCR tubes containing 200 µl of liquified, undiluted semen for 5 min to allow sperm to enter the capillary tubes. After 5 min, capillaries were removed, and the distance of the furthest migrating sperm (i.e. vanguard sperm) was measured under an inverted microscope (*t* = 5 min). Capillaries were then rested horizontally on a warming plate at 37°C for the subsequent 55 min, with vanguard sperm migration distance assessed at the indicated times via inverted microscopy. For assessing sperm migration in semen treated with sAC inhibitor to mimic on-demand male contraception, 200 µl of semen was pre-incubated with the sAC inhibitor TDI-11861 (Miller *et al.*, 2022) at 100 nM for 10 min prior to insertion of the media-filled capillaries. For assessing sperm migration in viscoelastic media containing sAC inhibitor to mimic vaginally delivered female contraception, capillaries were filled with media containing 100 nM TDI-11861. Technical replicates of semen samples from individual donors migrating through 1% MC (Supplementary Fig. S1) revealed that for every donor, assessing the distance traveled by the furthest migrating sperm (i.e. vanguard sperm) followed the same time dependency, validating the use of vanguard sperm as our measure of sperm migration.

### Data analysis

Data are shown as mean ± SD/SEM as indicated in the figure legend; n indicates the number of biological replicates. Figures were designed using CorelDRAW^®^ Graphics Suite 2022 (Corel Inc., Version 24.06.301 Austin, TX, USA), and statistical analysis was performed using GraphPad Prism (GraphPad, Version 10.2.3 San Diego, CA, USA) using paired *t*-tests/Wilcoxon-rank-tests.

## Results and discussion

CASA is routinely used to assess motility of sperm purified from semen and diluted in HTF. We compared this motility with the motility of unpurified sperm directly from semen or when incubated in viscoelastic media (Fig. 1; Supplementary Videos S1, S2, S3, S4, S5, S6, S7, and S8; Table 1). As expected, we observed a significant increase in progressive motility (Fig. 1A) and VCL (Fig. 1B) in purified sperm incubated in capacitating (CAP: + bicarbonate and HSA) medium compared to those in non-capacitating (NC) medium. We performed the same experiment with whole semen to examine motility of unpurified sperm (Fig. 1). As observed in the representative images from the CASA in Fig. 1D, unpurified sperm from semen were accurately detected and tracked; however, overall progressive motility was diminished relative to purified sperm, and the difference between NC and CAP was not significant (Fig. 1A). We also observed no capacitation-dependent change in VCL in semen samples (Fig. 1B).

**Table 1.** CASA (computer-assisted sperm analysis) motility parameters for isolated sperm/semen diluted in HTF (human tubal fluid) with and without 1% MC (methylcellulose) media at 1 h.*

	HTF				MC			
	NC sperm	CAP sperm	NC semen	CAP semen	NC sperm	CAP sperm	NC semen	CAP semen
**Total motility (%)**	59 (12)	75 (17)	59 (22)	63 (9)	37 (20)	30 (13)	60 (21)	48 (24)
**Progressive motility (%)**	30 (12)	51 (16)	24 (11)	32 (12)	16.3 (16)	15 (9)	1 (1)	5 (3)
**Hyperactivated motility (%)**	1 (1)	8 (11)	1 (1)	1 (2)	0 (0)	2 (4)	0 (0)	0 (0)
**VAP (µm/s)**	45 (13)	64 (18)	39 (12)	54 (24)	35 (13)	46 (14)	22 (8)	32 (5)
**VCL (µm/s)**	77 (20)	102 (28)	63 (26)	75 (26)	46 (14)	69 (24)	32 (9)	47 (7)

* The values represent the mean (SD) values for all experimental conditions for all experiments depicted in Fig. 1. Motility (total, progressive, and hyperactivated) are shown in percentage. Velocities (VAP and VCL) are depicted as absolute values.

NC, non-capacitating; CAP, capacitating; VAP, average path velocity; VCL, curvilinear velocity.

In order to assess whether sperm motility could be effectively assessed via CASA when incubated in viscoelastic media, we diluted both purified sperm and semen in HTF with 1% MC (Fig. 1E and G; Table 1). As expected, given the viscoelasticity challenge, motility and velocity decreased between aqueous (HTF) and viscoelastic media (MC) to the point where progressive motility showed no capacitation-dependent increase (in purified sperm) or was too low to make reliable interpretations (in semen). For VCL, there remained a bicarbonate-stimulated increase in viscoelastic media. Taken together, these data suggest that we are unable to assess motility of sperm from unpurified and undiluted semen samples in viscoelastic media via CASA during clinical development.

**Figure 2.**
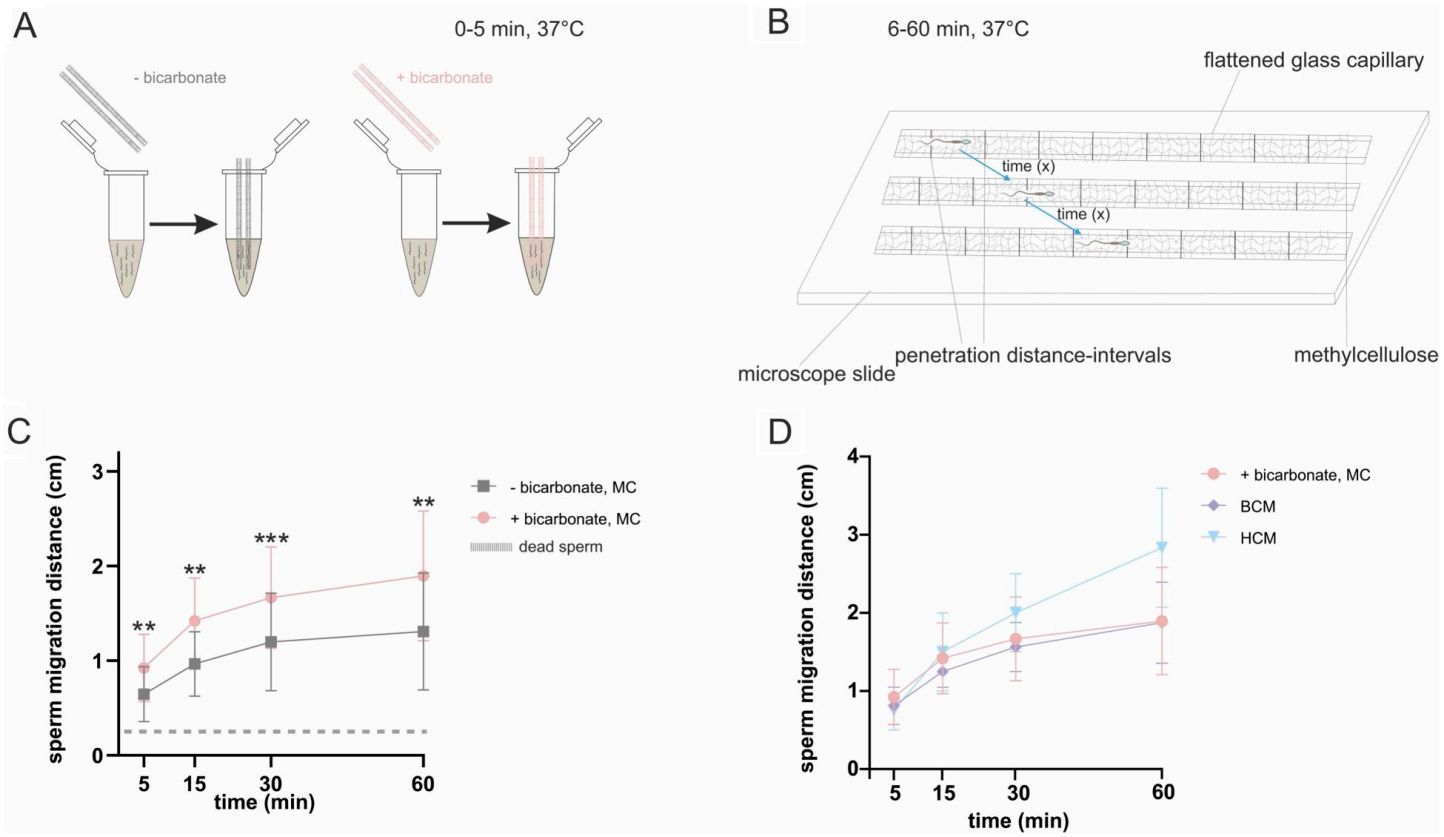
**Sperm migration in capillary assay.** (**A**) Schematic representation: glass capillaries were filled with methylcellulose (MC) in human tubal fluid (HTF) and then inserted into undiluted semen for 5 min. (**B**) Schematic representation: sperm migration within the capillaries was tracked over time (*t* = 6–60 min). (**C**) Migration of sperm in viscoelastic media (1% MC) in the absence (−bicarbonate) and presence (+bicarbonate) of bicarbonate. (**D**) Migration of sperm in viscoelastic media (MC, bovine cervical mucus, diluted human cervical mucus HCM). Data are shown as mean ± SD, representative of 3–12 independent experiments (different sperm donor samples). HCM was collected from one female donor. *P* value: **P < 0.01, ***P < 0.001 (paired *t*-test/Wilcoxon-rank-test). BCM, bovine cervical mucus; HCM, human cervical mucus; MC, methylcellulose.

We next explored whether sAC inhibitors affect sperm migration through viscoelastic media in a capillary migration assay (Fig. 2A and B). Sperm-mucus penetration tests in capillary tubes were shown to effectively correlate with IVF fertilization rates (Abu-Heija *et al.*, 1996). Sperm migrated from semen through MC-filled capillary tubes in a time-dependent manner (Fig. 2C). To ensure the migration distance was dependent upon real motility and not due to simple capillary action, we showed that dead sperm from heat-inactivated semen (60°C, 15 min) did not migrate further than 0.25 cm (Fig. 2C). As predicted, inclusion of bicarbonate in the MC-filled capillary tube to recapitulate capacitation conditions increased both migration speed and ultimate migration distance (Fig. 2C), confirming that sperm migration in viscoelastic media is stimulated by bicarbonate. We compared migration through MC with sperm migration in capillaries filled with either BCM or diluted HCM. In all cases, sperm migrated in a time-dependent manner (Fig. 2D), without significant difference in the migration speed or ultimate distance.

**Figure 3.**
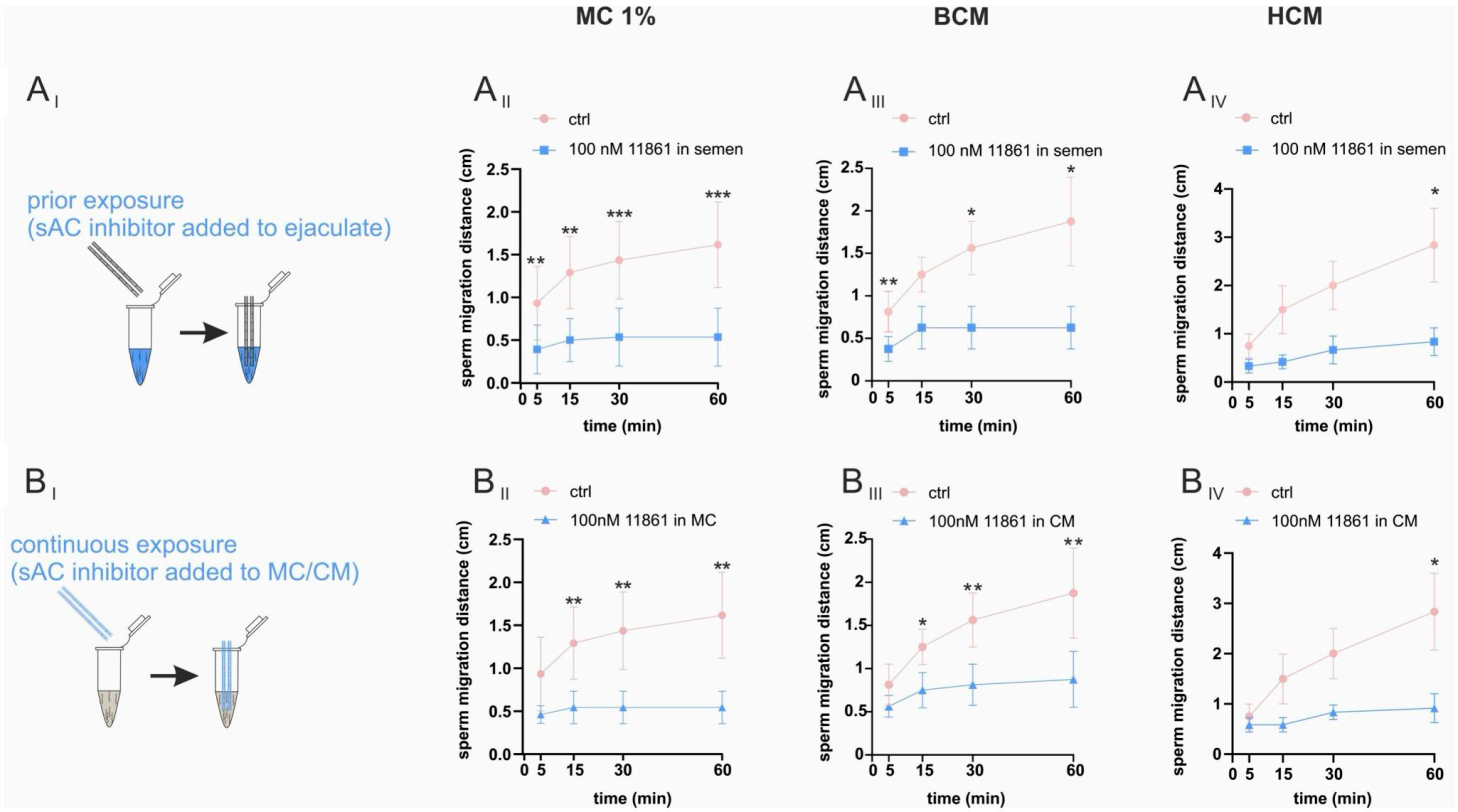
**Soluble adenylyl cyclase (sAC) inhibitors block sperm migration in methylcellulose (MC) and cervical mucus (CM).** (**A**) Sperm were pre-incubated with 100 nM TDI-11861, before introducing capillaries with 1% MC (n = 7 semen donors), bovine cervical mucus (BCM, n = 4 semen donors), or diluted human cervical mucus (HCM, n = 3 semen donors, diluted 1:3 in phosphate-buffered saline). Sperm migration was assessed under a microscope after 5, 15, 30, and 60 min. Data shown as mean ± SD. (**B**) For continuous exposure of sperm to sAC inhibitors, 100 nM TDI-11861 was added directly to the capillaries. *P < 0.05, **P < 0.01, ***P < 0.001 (paired *t*-test/Wilcoxon-rank-test).

We examined the efficacy of sAC inhibitors at blocking migration in 1% MC, BCM, or diluted HCM under conditions designed to mirror the conditions that will be used to study the contraceptive efficacy of sAC inhibitors delivered to either men or women (Fig. 3). We mimicked a sAC-targeted male contraceptive by preincubating semen with TDI-11861 for 10 min prior to allowing the sperm to enter the viscoelastic media-filled capillary (Fig. 3A_i_). Serum concentrations of TDI-11861 in excess of 1 µM proved efficacious during *in vivo* proof-of-concept experiments (Balbach *et al.*, 2023); however, compound concentrations in sperm and seminal plasma were not determined. Because concentrations of TDI-11861 in excess of 10 nM were sufficient to block hyperactivated motility and VCL in human sperm (Ritagliati *et al.*, 2025), we tested the efficacy of exogenous addition of 100 nM TDI-11861 in semen. This condition mimics an on-demand male contraceptive because the only sAC inhibitor present during migration will be the inhibitor prebound to sAC protein inside the sperm along with any inhibitor that diffuses into the mucus-filled capillary from semen. Thus, this treatment also recapitulates the dilution effect that happens to an on-demand male contraceptive taken by a man after ejaculation into a drug-free woman.

In all three conditions, MC, BCM, or HCM, sperm pretreated with 100 nM TDI-11861 failed to migrate further than 0.5 cm (Fig. 3A_ii_–A_iv_). These data demonstrate that inhibition of sAC activity in sperm, under conditions reflecting the mechanism of inhibition for an on-demand male contraceptive, prevents migration through viscoelastic media.

We also mimicked conditions to assess the potential of sAC inhibitors as a non-hormonal, female contraceptive by adding 100 nM TDI-11861 (Balbach *et al.*, 2023) directly to the capillaries filled with the three viscoelastic media (Fig. 3B_i_) to provide continuous exposure during migration. Similar to the situation where sperm were pre-exposed to TDI-11861, there was a significant decrease in migration in the presence of the sAC inhibitor in all three media (Fig. 3B_ii_–B_iv_). To test whether these effects were due to changes in the physicochemical properties of the media caused by a 100 nM inhibitor, we leveraged a less potent sAC inhibitor from the same chemical species. LRE1 is a less potent progenitor of TDI-11861 inhibiting sAC protein *in vitro* with an IC_50_ of 3 µM (Ramos-Espiritu *et al.*, 2016; Rossetti *et al.*, 2022). When LRE1 is included in the 1% MC at 100 nM, 30-fold below its IC_50_, it had no appreciable effect on entry into the capillaries, indicating that 100 nM small molecule addition does not adversely alter sperm migration in 1% MC (Supplementary Fig. S2). As expected, when included at 100 µM, 30-fold above its IC_50_, LRE1 prevented sperm entry, analogous to the effects of 100 nM TDI-11861. These data provide *ex vivo* proof-of-concept that a sAC inhibitor topically applied in the vagina, or systemically delivered with appropriate biodistribution into the female reproductive tract, can provide effective non-hormonal female contraception.

In addition to providing independent *ex vivo* confirmation that sAC inhibitors can provide effective contraception for men and women, these data confirm the utility of a sperm migration assay for examining contraceptive agents that affect sperm motility. Because this assay functions well with minimally manipulated, undiluted semen, it can be readily adapted for examining effective biodistribution into sperm and/or semen of an on-demand male sAC inhibitor contraceptive during the early phases of clinical trials and serve as a potential biomarker complementary to other sperm assays such as CASA.

## Supplementary Material

gaag035_Supplementary_Data

## Data Availability

The raw data obtained in this study are available upon request from the corresponding author.
