# Supplementary material for: Assessing efficacy of ADCY10 inhibitors in cervical mucus
Source: Mol Hum Reprod. 2026 Jun 4;32(2):gaag035. doi: 10.1093/molehr/gaag035 (PMC13275317; doi:10.1093/molehr/gaag035)
Supplement: gaag035_Supplementary_Data [file gaag035_supplementary_data.zip › 260728_Supplementary_Information.pdf]

Supplementary Information

Assessing efficacy of ADCY10 inhibitors in cervical mucus

Justine Fischoeder, Jai G. Marathe, Deborah J. Anderson, Lonny R. Levin, Jochen Buck, and Carla Ritagliati

Contents

Supplementary Figure S1: Technical replicates.

Supplementary Figure S2: Sperm entry into MC (methylcellulose)-filled capillaries.

Supplementary Videos S1-8: Exemplary computer-assisted sperm analysis (CASA) videos. *

*The supplementary videos have been provided as separate files.

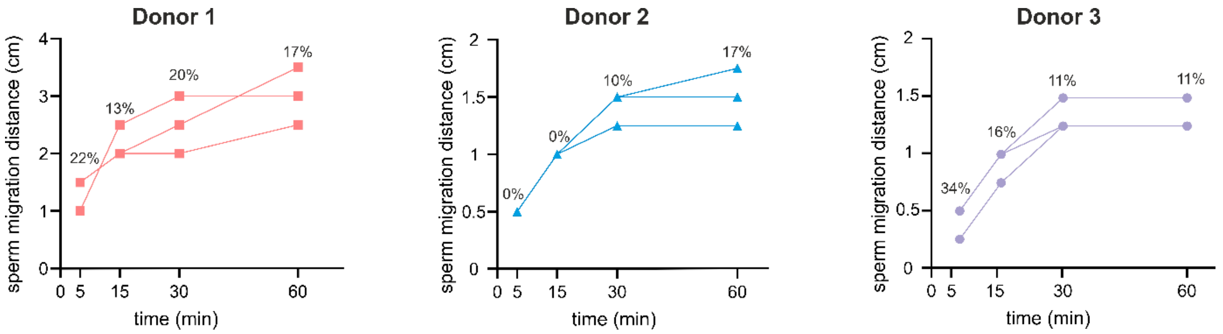

Supplementary Figure S1 Technical replicates. The depicted traces show three technical replicates from three individual donors (sperm samples). The semen was untreated, the methylcellulose contained bicarbonate and human serum albumin. Sperm migration was tracked over time. The coefficient of variation (CV) was calculated for all time points for all three donors as follows: $CV = \left(\frac{\text{Standard Deviation}}{\text{Mean}}\right) \times 100\%$. Donor 1: 22% (5 min), 13 % (15 min),20 % (30 min),17% (60 min); Donor 2: 0% (5 min),0% (15 min), 10% (30 min), 17% (60 min); Donor 3: 34% (5 min), 16 % (15 min),11% (30 min), 11% (60 min).

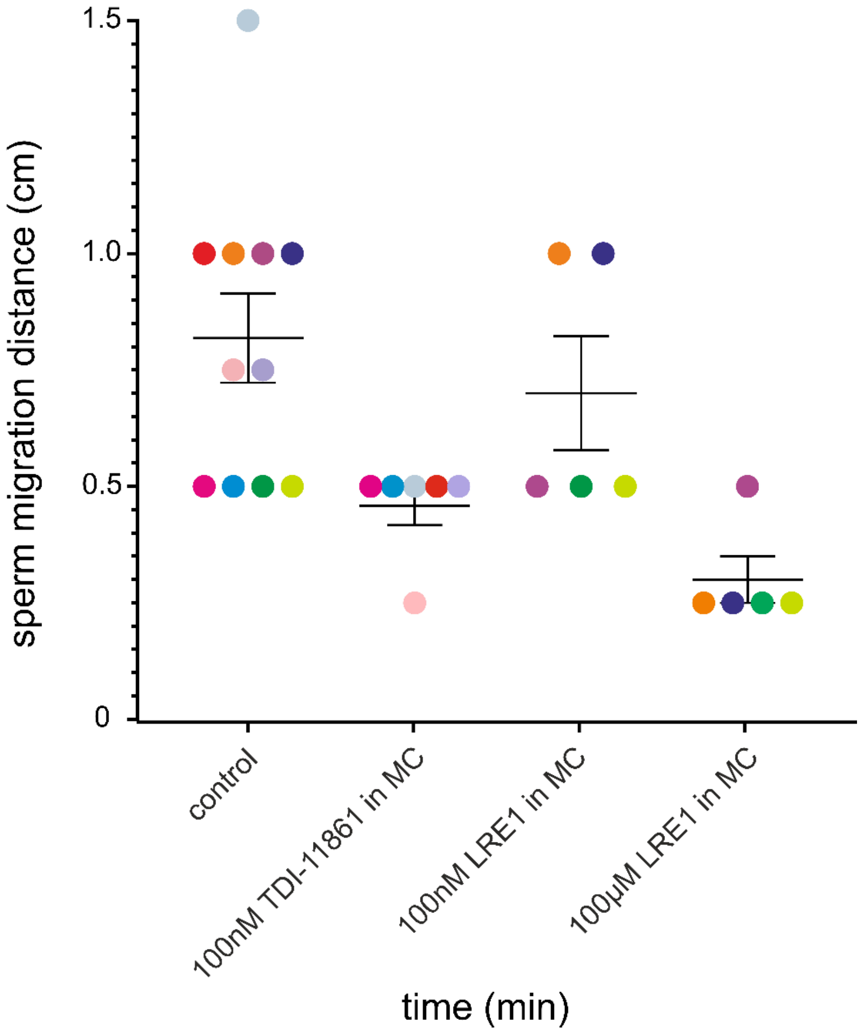

Supplementary Figure S2 Sperm entry into MC (methylcellulose)-filled capillaries. Data depicts sperm entry at 5 minutes under control (+bicarbonate, + human serum albumin) conditions compared to sperm entry with capillaries filled with methylcellulose containing either 100nM TDI-11861, 100nM LRE1 or 100µM LRE1. Data shows individual values, mean +/- SEM. Individual donors are color-coded (dots).

Supplementary Video S1 Exemplary computer-assisted sperm analysis (CASA) video. Sperm from human donors was diluted in human tubal fluid (HTF) under non-capacitating (NC) (-bicarbonate, human serum albumin) condition. Track‘s color code: motile (green), progressive (turquoise), hyperactivated (orange), slow (pink), static (red).

Supplementary Video S2 Exemplary computer-assisted sperm analysis (CASA) video. Sperm from human donors was diluted in human tubal fluid (HTF) under capacitating (CAP) (+bicarbonate, human serum albumin) conditions. Track‘s color code: motile (green), progressive (turquoise), hyperactivated (orange), slow (pink), static (red).

Supplementary Video S3 Exemplary computer-assisted sperm analysis (CASA) video. Semen from human donors was diluted in human tubal fluid (HTF) under non-capacitating (NC) (-bicarbonate, human serum albumin) condition. Track‘s color code: motile (green), progressive (turquoise), hyperactivated (orange), slow (pink), static (red).

Supplementary Video S4 Exemplary computer-assisted sperm analysis (CASA) video. Semen from human donors was diluted in human tubal fluid (HTF) under capacitating (CAP) (+bicarbonate, human serum albumin) condition. Track‘s color code: motile (green), progressive (turquoise), hyperactivated (orange), slow (pink), static (red).

Supplementary Video S5 Exemplary computer-assisted sperm analysis (CASA) video. Sperm from human donors was diluted in human tubal fluid (HTF) (+methylcellulose, MC) under non-capacitating (-bicarbonate, human serum albumin) condition. Track‘s color code: motile (green), progressive (turquoise), hyperactivated (orange), slow (pink), static (red).

Supplementary Video S6 Exemplary computer-assisted sperm analysis (CASA) video. Sperm from human donors was diluted in human tubal fluid (+methylcellulose, MC) under capacitating (+bicarbonate, human serum albumin) condition. Track‘s color code: motile (green), progressive (turquoise), hyperactivated (orange), slow (pink), static (red).

Supplementary Video S7 Exemplary computer-assisted sperm analysis (CASA) video. Semen from human donors was diluted in human tubal fluid (+methylcellulose, MC) under non-capacitating (-bicarbonate, human serum albumin) condition. Track‘s color code: motile (green), progressive (turquoise), hyperactivated (orange), slow (pink), static (red).

Supplementary Video S8 Exemplary computer-assisted sperm analysis (CASA) video. Semen from human donors was diluted in human tubal fluid (+methylcellulose, MC) under capacitating (+bicarbonate, human serum albumin) condition. Track‘s color code: motile (green), progressive (turquoise), hyperactivated (orange), slow (pink), static (red).
